# Louis Pasteur (1822–1895), Ignaz Semmelweis (1818–1865), Joseph Lister (1827–1912) and the Link Between Their Works Toward the Development of Antisepsis: A Narrative Review

**DOI:** 10.7759/cureus.62543

**Published:** 2024-06-17

**Authors:** Theocharis Lepenos, Despina Sanoudou, Athanase Protogerou, Konstantinos Laios, Georgios Androutsos, Marianna Karamamou

**Affiliations:** 1 History of Medicine and Medical Ethics, National and Kapodistrian University of Athens School of Medicine, Amfikleia, GRC; 2 Clinical Genomics and Pharmacogenomics Unit, 4th Department of Internal Medicine, Athens, GRC; 3 Clinic & Laboratory of Pathophysiology, National and Kapodistrian University of Athens, Athens, GRC; 4 Surgery, Department of History of Medicine and Medical Ethics, National and Kapodistrian University of Athens, Athens, GRC; 5 Department of History of Medicine and Medical Ethics, Medical School, National and Kapodistrian University of Athens, Athens, GRC; 6 Biomedical Research Foundation, Academy of Athens, Athens, GRC; 7 History of MEdicine and Medical Ethics, National and Kapodistrian University of Athens School of Medicine, Athens, GRC

**Keywords:** ignaz semmelweis (1818-1865), joseph lister (1827-1912), history of medicine, antisepsis, louis pasteur (1822-1895)

## Abstract

Infection control remains a significant burden for healthcare systems. The irrational use of antibiotics in the fight against microbial diseases has led to the fast development of antimicrobial resistance. Considering how the latter can adversely influence the effectiveness of modern treatments and the way medicine is practiced, we should revise the events that led to the establishment of the general principles of antisepsis and pay special tribute to the people who contributed to their formation, bearing in mind that they remain unmodified to a great extent until today. Without Semmelweis’ conceptualization of the idea of direct transmission of sepsis, Pasteur’s emblematic figure that helped promote the idea even further, and Lister’s methodology structuring, the scientific community would have significantly delayed winning the battle against germs.

## Introduction and background

Crucial role of antisepsis in the post-COVID-19 era

Undoubtedly, the outburst of the pandemic that recently impacted the whole of humanity radically reminded the medical community of the importance of meticulous implementation of antiseptic measures, virus transmission prevention measures, and the power of vaccinating the general population against an infectious disease. Nowadays, no one can question the value of all these techniques, but some centuries ago, when they were first introduced, they received harsh skepticism.

Antisepsis relates to the process of removing transient pathogens from any surface to an extent that infection can be prevented. A variety of techniques to destruct or inhibit viable microorganisms on any living tissues, the skin, the operating field, or the surgeon’s hands have been designed as prophylactic procedures to promote asepsis and eliminate the harmful results of contamination (bacterial, viral, fungal, and other) using sterilization and disinfection. Thorough antiseptic measures implementation is as crucial for overall high-standard patient care as a correct diagnosis or good surgical technique [1,2]. 

In particular, antiseptic methods were developed after the infectious nature of certain maladies was confirmed or at least hypothesized. Back in his time, Semmelweis suggested the most obvious, that everyone participating in a baby’s delivery should have previously washed their hands. However, until today when we use special substances and equipment to achieve high standards of antisepsis, infections remain an ongoing challenge. This is due to different factors compared to the situation back in the 19th century though. Despite the universal strict adherence to infection prevention, infections constitute a problem that is yet to be solved, since the percentage of anti-microbial resistance is at unprecedented heights [3].

Infection control is also at the center of the World’s Health Organization (WHO) current initiatives as reflected in its campaigns concerning hand hygiene, surgical site infection, sepsis, and antimicrobial resistance. This underlines the timeless combat of the healthcare systems against this threat to public health. Furthermore, this justifies the need to recall the innovators who supported for the first time the great potential of antisepsis in totally improving healthcare. They should not only be deemed as pioneers during their times but as highly influential scientists until today [4].

Relevant published literature was searched via PubMed for studies and historical references concerning various milestones during the life of the three prestigious scientists in question. Their crucial achievements in the biomedical field were reviewed. Historical events are chronologically placed to synthesize an even narration of the circumstances that led to the acceptance of the scientists’ conceptions. In particular, the authors focused on any potential interconnections between their scientific activities that were hence critically analyzed. Information was retrieved from manuscripts in both the English and the French language.

## Review

Semmelweis’ observation, hypothesis, and intervention

During the first decades of the 19th century, an obstetrician from Hungary named Ignaz-Philip Semmelweis (1818-1865) working in the Vienna General Hospital was the first to prove, based on experimental evidence, the contagious nature of puerperal fever, a type of sepsis adversely affecting women early in the period after delivery. He had observed that the increased incidence of this serious and potentially fatal condition was associated with medical students practicing in a ward with post-partum women. The students had previously come to contact barehanded with dead human bodies on which autopsies were performed for the purposes of anatomy learning. His observation led to the hypothesis that the hands of the students were responsible for the transmission of the disease.

There were two separate maternity wards in the hospital, the key difference between which, was the staff occupied in each ward. In the department with the lower mortality rate, the staff consisted solely of midwives, whereas the one with the higher rate, also received medical students right after conducting corpses’ dissections. Besides, medical students’ education through participation in deliveries was a priority passionately supported by Semmelweis' superior, Dr. Johann Klein (1788-1856), Head of the Maternity Sector [5].

However, the ambitious Ignaz, moved by the percentage of deaths in new mothers, wanted to alter this negative situation. Thus, he requested that the students meticulously wash their hands right after their anatomy sessions and before assisting with childbirth. This measure brought a tremendous result on the post-partum mortality rate (limiting it from 16% to the impressive 1%). Consequently, he proposed the basic principles of antisepsis and hand hygiene with the use of special solutions that could be used to get rid of the substance culprit for childbed fever.

And yet, the so-called “savior of post-partum women,” received harsh criticism from many of his colleagues, who questioned his observation, even years later. Despite the undeniably positive conclusion of his experiment and the deaths that had been prevented, the implementation of hygienic prophylaxis in healthcare settings was not universally accepted until much later. Not only did his theory not receive the recognition it deserved, but he was also dismissed from his hospital. He was forced to return to his homeland, where again he was not welcome. He started working at the Saint Rochus Hospital in Pest, and again by applying his methods, he managed to drastically reduce contamination and lower the mortality rates after labor to 1% [5,6].

Owing to the distrust from the medical community, the Hungarian doctor chose to publicly share his works in the Hungarian press only after 11 years. Two years later, he also published his theory in a book in the German language, where he gathered the stages of his experiment, statistical analysis, and figures. The following year, his book received criticism by Dr Eugène Follin (1823-1867) and Charles Lasègue (1816-1883) deeming his results as not insightful in terms of what they taught the medical community about puerperal sepsis, but only beneficial as far as the use of statistical terms and analytics is concerned. At the same time, they rejected his discoveries by intensively criticizing his character and practices regarding scientific dialogue as not constructive and rather unprogressive.

Ignaz Semmelweis's cause and nature of death at his 47 (1865) remains an unsolved mystery. According to the researcher Louis Ferdinand Céline, whose interest focused on the Hungarian doctor’s life, Ignaz died of sepsis, the disease he had devoted his life to cure after he committed suicide by harming himself using a scalpel previously utilized in a cadaver’s autopsy. In reality, his sepsis did not derive from his own hands, but from multiple, untreated traumas that occurred during his stay at an insane asylum.

Despite the significant delay of his publication, his disputant personality, and his tragic end, the spread of Ignaz’s ideas had started, as early as, 1848 when the French Academy of Sciences published a note of his in their weekly report, describing the new knowledge. As anticipated, the medical nomenclature of that time did not approve of his conclusions. This note, though, was the start of his work, to gain field and little by little get considered or even accepted by his colleagues [7].

Stages of Semmelweis’ ideas dissemination and acceptance

Luckily, many scientists after Semmelweis contributed to the gradual embracement of his theory. By the time of his discovery, in 1847, a friend of his and dermatologist at the General Hospital, Ferdinand von Hebra (1816-1880), informed the Viennese Society of Physicians about the cause of post-partum sepsis, which was nothing but an infection deriving from cadavers. The following year, Ignaz’s student Charles Henry Felix Routh (1822-1909), sent a letter to the medical-surgical society of London, where it was stated that hospitals also in Strasbourg and Prague were facing similar conditions. Furthermore, at a maternity ward in Kiel, Germany, Professor Gustav Adolf Michaelis (1798-1848) started applying Semmelweis' techniques, after reporting exceeding rates of mortality, and then surprisingly deaths dropped significantly. Unfortunately, he did not actually assign himself to the establishment of antisepsis, since he was overwhelmed by his thoughts and accused himself of so many preventable deaths, that he put an end to his life.

Semmelweis’ ideas were already becoming more and more popular, when in 1849, a Viennese renowned doctor and reformer of medical education, Joseph Skoda (1805-1881), announced his works to the Vienna Academy of Sciences. Although Semmelweis did not wholeheartedly encourage this intervention, he used it as a chance to reconcile with his former Chief in the Vienna General Hospital, Dr. Klein. Another supporter of Semmelweis’ theory was a former head of the maternity department in Vienna, Franz Hector Arneth (1818-1907). He presented Iganz’s conclusions twice in 1851 before the French Academy of Medicine and the Medico-Surgical Society of Edinburgh [8].

Contribution of Louis Pasteur

The French chemist Louis Jean Pasteur (1822-1895), a homo universalis of his time was the father of modern Microbiology (along with Robert Koch (1843-1910), a title he won thanks to his legacy of important achievements and discoveries. He conducted several experiments and proved that the cause of any infectious disease is a microorganism [9]. Thus, the hypothesis of spontaneous generation was debunked, after years of fighting against it, and the modern Germ Theory was globally adopted. Thanks to his reputation and wide approval in his era’s scientific circles, he managed to immensely influence this anachronistic, till that time, the field of science. Microbiology has made a big step forward [10].

He paved the way for the rather belated recognition of Semmelweis’s work when he discovered the hemolytic streptococcus as the cause of postpartum sepsis in 1879, leading to the definitive conclusion that so many young mothers could be saved and puerperal fever could be prevented, just by enforcing antiseptic techniques in maternity clinics [11].

Paradoxically, years later this new piece of life-saving knowledge was still not happily addressed by part of the medical community. In the year 1892, at the Paris Medical Academy, a famous gynecologist criticized the theory about the direct transmission of puerperal fever. Suddenly, someone in the audience interrupted him, rose to the podium, drew some pictures representing streptococci on the blackboard, and said in a soft voice: “Here is your infection, sir.” The gynecologist did not answer, because the gentleman who had taken the stage was Louis Pasteur [8].

Joseph Lister: the “architect” of antisepsis

The work of the eminent French chemist Louis Pasteur on fermentation and putrefaction proved that these biological processes are due to the action of microorganisms. Pasteur fought hard to prove his theory by conducting a series of experiments. He proved that the germs of the air are identical to those that cause the phenomena of fermentation and putrefaction and that they are destroyed by heat. He presented his results to the French Academy of Sciences and in 1861 published his well-known book entitled “Mémoires sur les corpuscules organizés qui existent dans l'atmosphère” [12].

At that time Joseph Lister (1827-1912), Professor of Surgery at the University of Glasgow, was facing a significant increase in mortality (45%) due to surgical wound infections. After all, infections and accompanying conditions (gangrene, septicemia, etc.) were major problems in surgery. Lister, studying the works of Pasteur, realized that inflammation and effusion were phenomena analogous to that of fermentation, due to microorganisms in the air, and the only definitive solution was the destruction of the microbes. As a method of disinfecting wounds, he used spraying with phenic acid, while codifying routine actions such as hand washing, cleaning the wound, applying a phenic acid-impregnated dressing, wrapping with waterproof material (thin tin foil), and recognizing the value of heat in sterilizing tools and clothing [13,14].

In 1867, he published his method in the medical journal The Lancet under the title “On the Antiseptic Principle in the Practice of Surgery” [15]. Although his method of antisepsis initially faced many critics, it proved to be a lifesaver during the Franco-Prussian War of 1870, saving thousands of lives. At that time he published his results on conducting amputations whilst implementing antiseptic measures (lowering mortality from 45% to 15%) and his methods gradually became widespread. Finally, it is worth mentioning the great friendship that connected the two gentlemen, Pasteur and Lister [16].

Nowadays the indisputable benefits of antisepsis that were highlighted by the works of Semmelweis, Pasteur, and Lister have led to the ongoing interest in up-to-date measures and policies in the direction of eliminating preventable infections, especially in the medical setting. Recently even more light was shed on the need for rigorous implementation of antisepsis techniques in order to avoid further transmission of a global health threat, a quite contagious virus. Therefore, a lot of research was conducted during and after the COVID-19 pandemic on newly developed disinfection procedures such as surface sterilization with the use of UVGI (Ultraviolet Germicidal Irradiation) devices, air-conditioning disinfection, specific chemical reagents, isolation policies for communicable diseases and revised transmission precautions.

Despite today’s normalization of the healthcare systems concerning SARS-COV-2, the problems of infection control and multi-drug resistant pathogens still constitute a huge challenge that needs to be addressed. Healthcare professionals should constantly stay vigilant for the adequacy of antisepsis when performing various procedures (hand hygiene, patient physical examination, invasive procedures, wound management, surgical site management, hospital facilities maintenance, etc.). One more time the answer lies in the examples of the three aforementioned prominent scientists who laid the foundations of contemporary microbiology [17-20].

## Conclusions

History has shown that many theories widely applicable in modern times, were intensively fought against when they were initially formulated. Moreover, several leading scientists such as Pasteur, father of microbiology, and Lister, founder of modern surgery and architect of antisepsis, and their predecessors such as Semmelweis confronted undeniable disapproval and even disrespect in their endeavor to modify clinical procedures toward better outcomes and less complications and to provide insight into complex medical issues. Nevertheless, their discoveries saved many lives and founded modern medicine, since many of the principles they introduced centuries ago are still in use assisting physicians in tackling real medical challenges of our times that affect patients worldwide. In particular, Louis Pasteur’s role in the dissemination of this knowledge proved to be definitive owing to his scientific prestige which ensured his approval amongst his colleagues.

In the period of rising antimicrobial resistance, it is pivotal to remind ourselves of the founders of antisepsis as a means to achieve infection control. Despite the evolutions in the biomedical field, infection control still constitutes a priority and an objective for the management of which all our forces should be joined.
